# Cycle Metrics and Strategy Detection for Automated Chair Sit-to-Stand Test Analysis Employing a Single Smartphone

**DOI:** 10.1007/s10439-025-03943-4

**Published:** 2025-12-21

**Authors:** Arshad Sher, Muntazir Rashid, Ahmad Lotfi, Federico Povina, Otar Akanyeti

**Affiliations:** 1 Department of Computer Science, Nottingham Trent University, Nottingham, NG11 8NS Nottinghamshire UK; 2 England, UK; 3 Department of Life Sciences, Aberystwyth University, Aberystwyth, SY23 3FL Ceredigion UK; 4 Department of Computer Science, Aberystwyth University, Aberystwyth, SY23 3DB Ceredigion UK

**Keywords:** Wearable sensors, Chair sit-to-stand (CST), Rising strategy classification, Functional mobility, Rehabilitation engineering, Parkinson’s disease

## Abstract

****Purpose**:**

The 30-second Chair Sit-to-Stand Test (30 s CST) is widely used to assess lower-limb function and reflects complex motor coordination across neural systems. However, conventional scoring methods are often inconsistent and fail to capture variations in compensatory movement strategies or require invasive instrumentation. This study presents a smartphone-based system that automatically detects rising strategies across repeated CST cycles, providing an automated approach to extract cycle-level biomarkers of motor performance.

****Methods**:**

Thirty-five adults 10 younger, 20 older, and 5 with Parkinson’s disease performed supervised 30-s CST trials while wearing a waist-mounted smartphone that recorded accelerometer and gyroscope data at 400 Hz. Cycle detection used amplitude-adaptive thresholds and dominant-frequency intervals for robust segmentation of CST cycles. Rising strategies were classified with rule-based method that uses trunk pitch dynamics and cycle duration. Agreement with video annotations was assessed using Intraclass Correlation Coefficients (ICC (2, 1)), Bland–Altman analysis, and macro F1 scores.

****Results**:**

The algorithm detected 660 CST cycles with 99% accuracy, and the average mean absolute error across participants was under 40 ms. Bland–Altman analysis showed negligible bias (− 0.012 s) and narrow limits of agreement (− 0.134 to 0.110 s). Strategy classification achieved macro F1 = 0.94. Flexion cycles were consistently longer than Momentum Transfer cycles (e.g., older adults: 2.63 vs. 1.45 s).

****Conclusion**:**

Automated CST analysis reveals movement signatures not captured by standard timing, offering a richer characterization of mobility patterns. While these findings demonstrate technical feasibility and highlight clinically relevant variations, their application for diagnostic or personalized rehabilitation purposes remains preliminary and requires validation in larger cohorts.

## Introduction

Functional mobility is a cornerstone of independence and quality of life, particularly in older adults and individuals with neuromuscular or musculoskeletal conditions [1–3]. Among clinical mobility assessments, the Chair Sit-to-Stand Test (CST) is widely adopted for its simplicity, minimal equipment requirements, and strong predictive validity for fall risk, frailty, and functional decline [4–7]. CST performance reflects lower-limb strength, postural control, and endurance and correlates with physical fitness and cognitive status in aging populations [6, 8, 9].

**Fig. 1:**
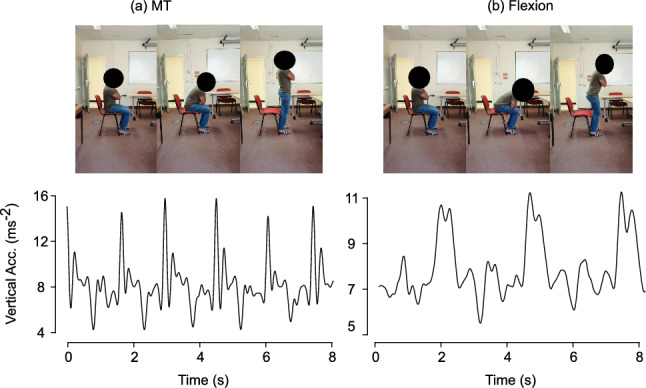
Chair rising strategies and vertical acceleration profiles: **a** MT with shorter cycle time. **b** Flexion with prolonged forward lean and fewer repetitions.

CST is typically administered as either the number of repetitions completed in a fixed time (e.g., 30 s, hereafter 30-s CST) or the time required to complete a fixed number of Sit-to-Stand (STS) repetitions (e.g., 5$\times$STS or 10$\times$STS) [8, 10–13]. Higher repetition counts or shorter completion times indicate better functional status. However, stopwatch-based scoring and visual observation are prone to inter-rater variability and fail to capture cycle level (i.e., the repetitions done over the time in CST or STS) dynamics or compensatory strategies (i.e., variation in rising from a seated position), limiting clinical interpretability and sensitivity to early mobility decline [14–17]. Beyond repetition counts, the way an individual rises from a seated position provides insight into neuromuscular control and compensatory mechanisms. Two predominant strategies are observed [18] as shown in Fig. 1: Momentum Transfer (MT), characterized by coordinated forward trunk inclination and rapid hip–knee extension, enabling efficient use of momentum; and Flexion Strategy, involving prolonged trunk flexion before extension, often adopted by frail individuals or those with reduced strength. These strategies influence cycle duration, trunk kinematics, and energy cost, and their identification can inform targeted interventions [19–21].

Existing literature on CST quantification has evolved from foundational clinical validation to instrumented sensing and, more recently, smartphone and video-based solutions that preserve biomechanical detail. The clinical foundation was laid by Jones et al., who introduced the 30-s CST as a reliable measure of lower body strength in older adults, reporting test–retest reliability of approximately 0.84 and normative values of 12–17 repetitions for women and 14–19 for men [6]. Early work demonstrated that single-sensor inertial approaches reduce setup burden and can be deployed in both clinical and home environments. Najafi et al. attached a gyroscope to the chest to measure sit-to-stand and stand-to-sit durations, validating against Vicon motion capture with strong correlation ($r = 0.9$) and linking prolonged transitions ($\ge 3.8 \pm 1.1$ s) to elevated fall risk [22]. Van Lummel et al. advanced instrumented STS analysis, showing that sensor-derived metrics associate more strongly with health status than manual timing [23, 24], while Atrsaei et al. demonstrated that kinetic and smoothness features outperform total time in predicting serious falls [25]. Pham et al. validated a lower-back IMU algorithm for postural transitions in Parkinson’s disease and older adults under home-like conditions, reporting high detection accuracy and clear group differences [26]. Park et al. explored frailty assessment using 5$\times$STS with multiple IMUs, finding significant differences in cycle times between robust and frail participants [27].

Smartphone-based approaches offer scalability and have shown high accuracy for repetition counting and timing. Marques et al. positioned a smartphone at the waist to capture repeated sit-to-stand cycles, achieving 94.4% accuracy and strong correlation with video [28]. Cobo et al. developed an app for 30 s CST, achieving ICC > 0.90 vs manual counts and demonstrating feasibility for home monitoring [29]. Cerrito et al. validated a smartphone app for single STS timing in healthy seniors [30]. Recent work by Sher et al. proposed a smartphone-based pipeline that computes CST scores and classifies rising strategies using machine learning, achieving >95% classification accuracy and mean absolute error <60 ms for cycle timing [31].

Despite these advances, most systems report only global outcomes and exclude features needed to interpret motor strategies (e.g., trunk pitch, peak accelerations) [29, 30]. Cycle-by-cycle detection often lacks subject-specific frequency adaptation, making algorithms vulnerable to spurious peaks in older adults or those with neurological conditions [32]. Several studies rely on dedicated sensors or controlled lab setups, limiting scalability and ecological validity [22–24, 26, 29]. These gaps motivate the present work: a single-smartphone, context-aware pipeline that delivers automatic cycle-level metrics and rising strategy classification. It may allow clinicians to identify cycle-specific impairments in Parkinson’s disease, young and older adults, supporting precise diagnosis, personalized rehabilitation, and continuous monitoring of mobility decline.

This study introduces a smartphone-based CST analysis pipeline that detects cycles using adaptive thresholds, extracts temporal and kinematic metrics, and classifies rising strategies using trunk pitch angle. Outputs are validated against video annotations using intraclass correlation coefficients (ICC). The approach enables detailed mobility assessment using only a consumer-grade smartphone, improving scalability and clinical interpretability.

## Materials and Methods

**Fig. 2:**
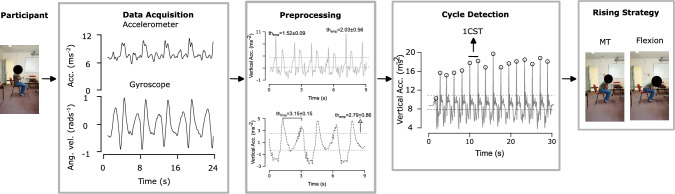
Framework of the proposed CST quantification pipeline illustrating sequential stages: participant recruitment and CST protocol, data acquisition, preprocessing (mean±std), and cycle detection/rising strategy classification.

To automate CST analysis, we implemented a structured pipeline as shown in Fig. 2, comprising participant recruitment, data acquisition, preprocessing, cycle detection, and strategy classification. Each stage is described in the following subsections.

### Participants

Thirty-five adults participated 10 younger (<60 years), 20 older (>60 years), and 5 with Parkinson’s disease (PD). All recruited participants provided written informed consent and were able to perform the 30-s CST unaided. The sample sizes were determined by recruitment feasibility and the study’s aim to prioritize older adults ($n = 20$), where chair-stand performance is most clinically relevant. Young adults ($n = 10$) were included as a baseline reference, and the PD sub-group ($n = 5$) was added to test feasibility in a clinical cohort. The findings for the PD sub-group should be interpreted as preliminary and not generalized beyond this small sample. These allocations were driven by feasibility and the exploratory nature of the study, rather than for statistical group comparisons. The study was approved by the University Research Ethics Committee and adhered to the Declaration of Helsinki.

Participants performed a standardized 30-s CST in a controlled laboratory setting. They were instructed to rise from a standard height chair as many times as possible within 30 s, with arms crossed over the chest to minimize upper limb assistance. A clinical exercise physiologist supervised all trials to ensure safety and protocol adherence.

### Data Acquisition

A Google Pixel 6a smartphone was secured at the lower back (L3–L5) using an adjustable belt pouch to maintain consistent orientation. The device recorded tri-axial accelerometer and gyroscope signals at 400 Hz. Sensor axes were aligned as follows: *x* (vertical), *y* (mediolateral), and *z* (antero-posterior). Synchronized video (GoPro Hero 6, 60 fps) served as ground truth for repetition counting and rising strategy classification.

Gold standard systems such as force plates and GAITRite offer high accuracy but are costly, complex, and restricted to laboratory settings [33, 34]. Video-based observation provides a practical, low-cost alternative suitable for real-world environments, aligning with our long-term goal of outdoor applicability. All CST trials were annotated under expert supervision to ensure precise temporal accuracy for event identification. Videos were annotated frame by frame by a single researcher with two years of experience in gait video analysis and subsequently reviewed by an exercise physiologist with over 20 years of clinical experience. The videos were recorded at 240 frames per second, providing high temporal resolution and minimizing the margin of error compared to lower frame rates, where critical transitions might be missed. Although inter-rater reliability was not calculated, given the study’s focus on smartphone validation rather than video annotation variability, a two-step review process was implemented to ensure annotation integrity.

### Preprocessing

We first attenuate high-frequency noise while preserving the spectral band that characterizes voluntary STS dynamics to ensure that subsequent peak detection and strategy classification only operate on movement relevant data. Raw tri-axial accelerometer $\{a_x(t),a_y(t),a_z(t)\}$ ($\text {m/s}^2$) and gyroscope $\{\omega _x(t),\omega _y(t),\omega _z(t)\}$ (rad/s) signals, sampled at 400 Hz, are filtered with a zero-phase, second-order Butterworth low pass filter at 6 Hz [35, 36]. “Zero phase” indicates forward backward application, preventing temporal distortion of peaks and event timings. A 6-Hz cutoff is chosen because STS movements predominantly occupy $<5$ Hz. We set the passband edge at 6 Hz to preserves voluntary trunk and pelvis dynamics [37, 38]. We chose a second-order Butterworth filter because it reduces unwanted high-frequency noise without distorting the main movement signals, keeping the peaks clear for accurate cycle detection and ensuring stable gyroscope calculations for rising strategy detection. Zero-phase filtering was applied to prevent temporal distortion of peaks, ensuring accurate event timing. The 6-Hz cutoff was selected based on prior biomechanical studies showing that voluntary sit-to-stand movements predominantly occur below 5 Hz.

### Cycle-Level Detection and Rising Strategy

CST involves coordinated motion across the sagittal and frontal planes and the smartphone’s body fixed axes can vary slightly between trials. The calculation of acceleration magnitude $(a_{\text {mag}}(t))$ starts with

1 $$\begin{aligned} a_{\text {mag}}(t) = \sqrt{a_x(t)^2 + a_y(t)^2 + a_z(t)^2}, \end{aligned}$$

where $a_x(t)$, $a_y(t)$, and $a_z(t)$ denote the vertical, mediolateral, and antero-posterior axes ($\text {m/s}^2$), respectively. The objective of this step is to mitigate axis misalignment and posture-dependent orientation changes so that large impulses from sit-to-stand and stand-to-sit phases are preserved irrespective of the phone’s exact tilt. Further, we use the Euclidean norm to reduce the risk of over reliance on a single axis (e.g., the vertical channel), otherwise we would miss movement components redistributed into the other axes during CST, especially for participants with chronic conditions.

We detect cycle defining peaks with constraints that reflect plausible human movement by combining movement acceleration into $a_{\text {mag}}(t)$. Before introducing the peak rules, we prepare two adaptive thresholds: one on amplitude to reject small adjustments and one on timing to prevent false positive repeats. We define an adaptive amplitude threshold ($th_{\text {amp}}$ ($\text {m/s}^2$)) of $a_{\text {mag}}(t)$:

2 $$\begin{aligned} th_{\text {amp}}&= \alpha \!\left( \max \nolimits _t a_{\text {mag}}(t) - \min \nolimits _t a_{\text {mag}}(t)\right) , \end{aligned}$$

where $th_{\text {amp}}$ ($\text {m/s}^2$) varies with each subject’s and $\alpha$ $\in$ [0.1,0.4]. The constant $\alpha$ controls sensitivity: smaller values are more permissive (risking false positives from posture shifts), while larger values are stricter (risking missed low amplitude but valid transitions). We set a default $\alpha {=}0.2$ based on pilot sensitivity analysis showing a favorable trade-off between missed and spurious detections, and we report robustness $\alpha \in [0.1,0.4]$ to acknowledge inter-individual variability. Equation 2 shows the adaptive amplitude threshold scales to each participant’s movement range, improving robustness across cohorts with different strength levels. This adaptive construction allows the same rule to function across younger, older, and PD cohorts with differing movement amplitudes.

After $a_{\text {mag}}(t)$, a subject-specific time threshold ($th_{\text {time}}$ (s)), is defined. It has a minimum inter-peak interval via the dominant frequency (DF) of $a_{\text {mag}}(t)$:

3 $$\begin{aligned} th_{\text {time}} = \frac{1}{DF}, \end{aligned}$$

where *DF* (Hz) is estimated using Welch’s method [39]. Welch’s method segments the signal, applies windowing, computes modified periodograms, and averages them to reduce variance relative to a single periodogram, providing a robust estimate of the primary repetition rate even in the presence of noise. The dominant-frequency guard enforces physiologically plausible timing by rejecting peaks occurring faster than the subject’s estimated repetition rate (see Fig. 4, Table 2). The resulting $th_{\text {time}}$ (s) enforces physiologically plausible timing by restricting peaks that occur faster than a typical cycle for that subject, which prevents false counting within a single transition and stabilizes detection across heterogeneous performance levels.

With these adaptive thresholds, we introduce the peak acceptance rule. A candidate peak at time $t_k$ is accepted if

4 $$\begin{aligned} a_{\text {mag}}(t_k) \ge th_{\text {amp}} \quad \text {and} \quad (t_k - t_{k-1}) \ge th_{\text {time}}, \end{aligned}$$

where $t_k$ and $t_{k-1}$ (s) are the timestamps of the current and previous accepted peaks, respectively. This rule integrates amplitude (strength of movement) and timing (rate of repetitions) to mark cycle boundaries that correspond to sit-to-stand or stand-to-sit events. Once peaks are established, cycle duration follows naturally as follows:

5 $$\begin{aligned} T_i = t_{k+1} - t_k, \end{aligned}$$

where $T_i$ (s) is the *i*-th full-cycle time. $T_i$ becomes both an outcome metric and a feature that informs strategy classification (details in Sect. 2.5), since MT executions tend to be faster than flexion dominant ones.

To distinguish rising strategies, we now complement the acceleration derived timing with gyroscope-derived posture change because acceleration alone cannot reliably quantify trunk pitch.

### Rising strategy classification and role of gyroscope

The change in trunk pitch within each cycle was estimated by integrating the angular velocity about the mediolateral axis, which represents the pitch axis in the sagittal plane. Let $\omega _y(t)$ (rad/s) denote the gyroscope signal aligned with the smartphone mediolateral axis at the waist; we compute

6 $$\begin{aligned} \Delta \theta _i = \int _{t_i^{\text {start}}}^{t_i^{\text {end}}} \omega _y(t)\,dt, \end{aligned}$$

where $\Delta \theta _i$ (rad) is the net pitch change over the *i*-th cycle, and $t_i^{\text {start}}$ and $t_i^{\text {end}}$ are its start and end times as defined by the accepted peaks. The gyroscope angular velocity captures trunk rotation without gravitational influence and is applied over short windows defined by detected events, effectively limiting drift.

Finally, strategy labels combine temporal and angular insights to detect:

7 $$\begin{aligned} \text {Strategy}_i = {\left\{ \begin{array}{ll} \text {MT}, & T_i < \tau _g \wedge \Delta \theta _i > \theta _{\text {thresh}}, \\ \text {Flexion}, & \text {otherwise}, \end{array}\right. } \end{aligned}$$

where $\tau _g$ (s) is an age-adjusted time threshold and $\theta _{\text {thresh}}$ (rad) is an empirically tuned pitch change threshold. The age adjustment in $\tau _g$ reflects known slowing of functional transitions with age and accommodates cohort differences (e.g., older adults or PD) so that “fast” cycles are interpreted relative to appropriate expectations. The choice of $\theta _{\text {thresh}}$ ensures that cycles labeled as MT exhibit a meaningful forward pitch excursion characteristic of leveraging momentum, whereas smaller pitch changes and/or longer cycle times are consistent with a flexion dominant strategy. Together, $T_i$ (from robust, DF threshold-based peak timing) and $\Delta \theta _i$ (from appropriate gyroscope axis integration) provide complementary evidence: time captures execution speed and pitch change captures movement strategy, yielding a classification that is both biomechanically interpretable and resilient to sensor orientation variability.

*Threshold Selection:* Initial time thresholds ($\tau _g$) were derived from earlier studies [40]. These thresholds were tuned through pilot sensitivity analysis and subsequently validated on the current dataset (see Fig. 7), confirming their robustness across participant groups without requiring retuning. These values were then validated across all participant groups without further adjustment. Final thresholds were set to 1.2 s for young adults, 1.6 s for older adults, and 2.0 s for participants with Parkinson’s disease (PD). These group-specific values reflect observed differences in sit-to-stand timing and align with biomechanical ranges reported in prior gait studies [26, 28]. The angular threshold ($\theta _{\text {thresh}}$) was fixed at 0.35 rad ($\approx 20^\circ$) across all groups, consistent with literature indicating this angle as a critical biomechanical requirement for sit-to-stand initiation [41]. Combining angular and temporal thresholds improves detection accuracy by accounting for both posture and timing dynamics, reducing false positives while maintaining generalizability. These thresholds were chosen to minimize overfitting and may require adjustment for larger or more diverse cohorts.

### Validation and Statistical Analyses

We employed a statistical framework designed to account for the hierarchical structure of the data, where individual CST cycles are nested within participants. Recognizing that treating the cycle-level observations as independent measurements violates independence assumptions and inflates significance estimates. To strictly satisfy the independence assumption, the primary inferential analysis (including correlations, *t* tests, and standard Bland–Altman analysis) was performed on participant-level aggregated data ($n=35$). For this primary agreement assessment, the mean cycle time was computed for each participant using both the smartphone-derived and video-annotated measurements. Agreement was quantified using Pearson (*r*) and Spearman ($\rho$) correlation coefficients to assess linearity and rank order, a Paired *t* test to check for systematic bias, Bland–Altman analysis to visualize the spread of differences and calculate Limits of Agreement (LoA), and the Intraclass Correlation Coefficient (ICC(2, 1)) (two-way random effects, absolute agreement) to quantify reliability. To utilize the full resolution of the dataset while respecting the nesting structure, a Linear Mixed Model (LMM) was fitted. The LMM modeled the difference between methods ($d_{ij}$) for the *j*-th cycle of the *i*-th participant as $d_{ij} = \beta _0 + u_i + \varepsilon _{ij}$, where $\beta _0$ represents the fixed bias, $u_i \sim N (0, \sigma ^2_{between})$ is the random intercept for the participant, and $\varepsilon _{ij} \sim N (0, \sigma ^2_{within})$ is the residual error. This approach allows for the calculation of nesting-aware LoA and tests for systematic method effects. Strategy comparisons (Flexion vs. MT) and group comparisons were primarily conducted using participant-level means to ensure valid inference, although cycle-level comparisons were reported only as exploratory sensitivity analyses.

## Results

All analyses were performed offline. Each 30-s trial required less than 1 s of processing per participant on a 2017 MacBook Pro (Intel i5, 8 GB RAM), indicating low computational demand. We begin by examining the distribution of the rising strategy, followed by the accuracy of CST cycle detection. Next, differences in rising strategies across groups are reported, and then agreement is assessed using Bland–Altman analysis. Finally, we conclude with a sensitivity analysis to evaluate the robustness of our algorithm.

A total of 660 CST cycles were analyzed across 35 participants, where 238 cycles (36.1%) were classified as Flexion and 422 cycles (63.9%) as MT. Strategy distribution varied by cohort: older adults contributed 168 Flexion and 285 MT cycles; younger adults 21 Flexion and 123 MT cycles; and participants with Parkinson’s disease (PD) 49 Flexion and 14 MT cycles. The algorithm detected the 660 CST cycles with 99% accuracy and a mean absolute error of less than 40 ms. Strategy classification achieved a high Macro F1 score of 0.94. Exploratory cycle-level analysis confirmed that Flexion cycles were significantly longer than Momentum Transfer (MT) cycles ($p = 0.0014$). These temporal differences showed that Flexion was consistently 52–81% slower than MT within each cohort (e.g., older adults: Flexion $2.63 \pm 0.80$ s vs MT $1.45 \pm 0.40$ s). At the participant level, Flexion tended to be slower than MT in older adults, but this difference did not reach significance (p = 0.285), likely reflecting the modest sample size.

**Fig. 3:**
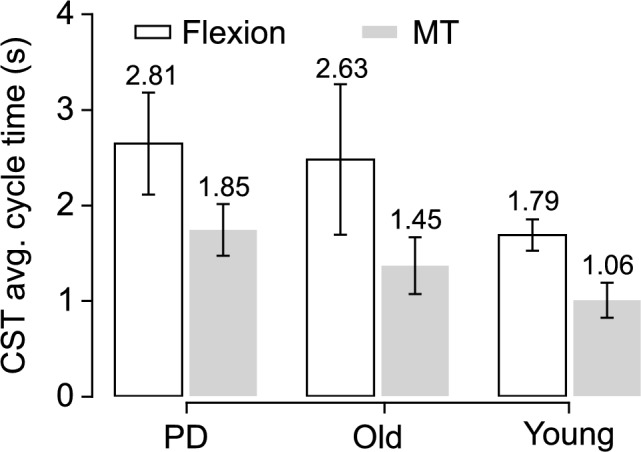
Average CST cycle time for MT and Flexion strategies across participant groups (PD, older, younger). Flexion cycles were consistently longer than MT cycles within each group.

Figure 3 demonstrates that flexion cycles were consistently longer than MT cycles across all groups. For PD participants, Flexion averaged 2.81 vs 1.85 s for MT; older adults averaged 2.63 s vs 1.45 s; and younger adults averaged 1.79 s vs 1.06 s. These differences correspond to Flexion being 52–81% slower than MT within each group. Younger adults completed more repetitions and exhibited shorter cycle times than older adults and PD participants.

**Fig. 4:**
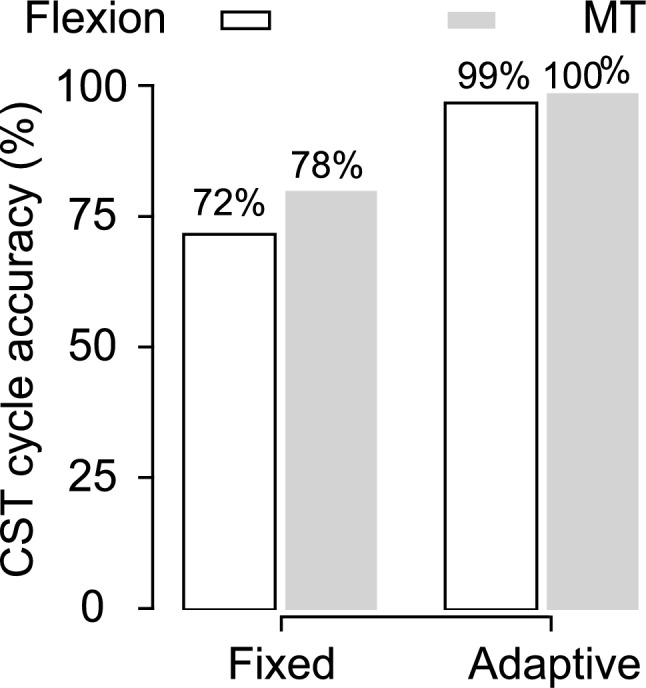
Demonstration of fixed vs adaptive threshold for detecting CST cycles in a trial where the rising strategy was either (a) Flexion strategy or (b) MT strategy across young, old, and PD participants.

Figure 4 shows the sensitivity analysis of the context-aware peak detection algorithm. Without adaptive thresholds, detection accuracy was 72% for Flexion and 78% for MT. Incorporating amplitude- and dominant frequency (DF)-based time constraints improved accuracy to 99% for both strategies. The summary of the results is given in Table 2. The average mean absolute error (MAE) in cycle time estimation across all groups was less than 40 ms, with group-specific values of 24.4 ms (younger adults), 31.4 ms (older adults), and 60.5 ms (PD).

**Fig. 5:**
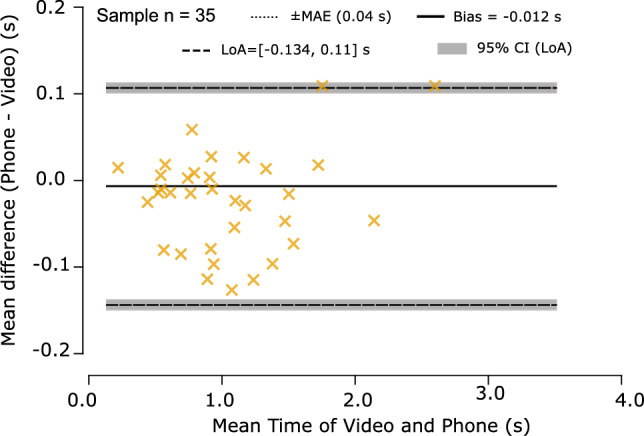
Bland–Altman plot comparing smartphone-based and video-based cycle time estimates across all participants (n = 35). The solid line indicates the mean difference (bias = − 0.012 s); dashed lines denote the 95% limits of agreement (LoA = − 0.134 s to 0.11 s). Shaded bands show the 95% confidence intervals of the LoA.

*Agreement and Reliability* The primary validation, performed on participant-level means, revealed near-perfect agreement between the smartphone and video-based scoring. The data showed a strong linear relationship (Pearson $r = 0.998, p < 0.001$) and robust rank order agreement (Spearman $\rho = 0.992, p < 0.001$). A paired *t* test on the participant means demonstrated no statistically significant difference between the methods ($t = -0.833, p = 0.416$), indicating an absence of systematic bias. This finding was supported by the participant-level Bland–Altman analysis (see Fig. 5), which showed a negligible mean bias of $-$0.012 s. The ICC(2, 1) for participant means was 0.998, demonstrating excellent reliability, and the MAE for participant-level estimation was approximately 36–40 ms, which is less than $2\%$ of a typical cycle duration.

At the cycle level, the LMM estimated a fixed bias of $-$0.007 s. The LMM confirmed that there was no significant method effect ($\beta _{phone-video} = -0.0065$ s, $p = 0.693$), verifying the robustness of the smartphone system when analyzing raw cycle data. The variance components quantified were $\sigma _{between} = 0.018$ s and $\sigma _{within} = 0.209$ s.

**Fig. 6:**
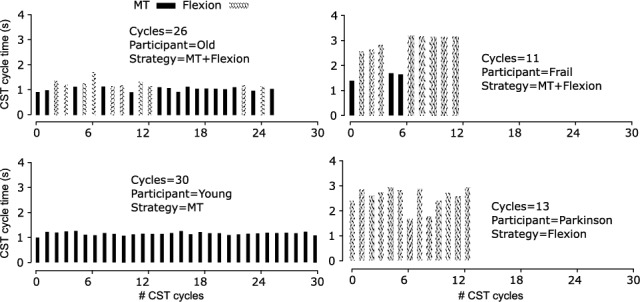
Examples of participants switching between MT and Flexion strategies within a single CST trial. Panels (a)–(d) show cycle-by-cycle classification and timing variability across PD, older, and younger adults.

Figure 6 illustrates trials where participants switched strategies mid-test, primarily among older adults. Table 1 summarizes all cycle-level metrics, including sit-to-stand and stand-to-sit durations, full-cycle time, repetition counts, and kinematic ranges for each cohort and strategy.

**Table 1:** CST performance metrics across participant groups (PD, older, younger) and rising strategies (Flexion vs. MT)

Metric	PD		Older		Younger	
	Flexion	MT	Flexion	MT	Flexion	MT
Sit-to-stand time (s)	$1.42\pm 0.30$	$0.95\pm 0.20$	$1.35\pm 0.40$	$0.81\pm 0.20$	$1.02\pm 0.20$	$0.65\pm 0.10$
Stand-to-sit time (s)	$1.39\pm 0.30$	$0.90\pm 0.20$	$1.28\pm 0.40$	$0.78\pm 0.20$	$0.97\pm 0.20$	$0.61\pm 0.10$
Full cycle (s)	$2.81\pm 0.60$	$1.85\pm 0.30$	$2.63\pm 0.80$	$1.45\pm 0.40$	$1.79\pm 0.20$	$1.06\pm 0.20$
Cycles (count)	$10.3\pm 2.4$	$15.5\pm 2.5$	$9.5\pm 2.4$	$20.3\pm 3.9$	$16.6\pm 1.7$	$27.0\pm 4.6$
Vertical acc. (m/s^2^)	($-2.2$, 10.6)	($-1.6$, 15.9)	(3.8, 17.5)	(1.7, 22.3)	(1.8, 20.1)	(2.1, 22.8)
Trunk ang. vel. (rad/s)	($-0.5$, 0.6)	($-0.4$, 0.3)	($-0.7$, 0.5)	($-0.5$, 0.4)	($-1.1$, 0.7)	($-0.3$, 0.4)

Values are mean ± SD. Acceleration and angular velocity ranges are reported as (min, max)

**Fig. 7:**
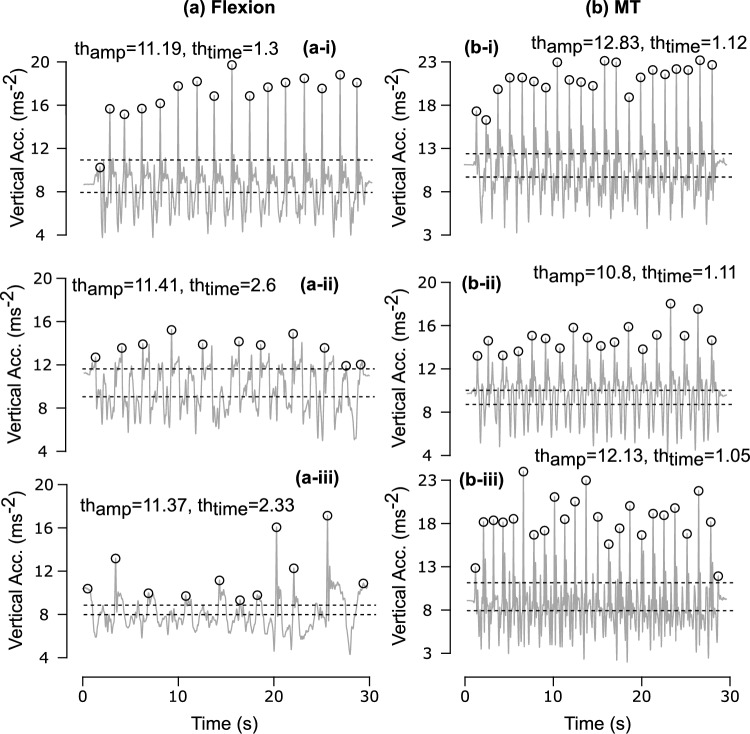
Sensitivity analysis illustrating the impact of algorithm components on cycle detection accuracy and timing precision: **a** Flexion strategy across older and PD participants; **b** MT strategy across young and older participants. Vertical acceleration profiles show threshold adaptation under different configurations.

Introducing the DF threshold increased detection accuracy from 72% to 99% (see Fig. 4). The importance of frequency-domain constraints is shown in Fig. 7 where amplitude and time thresholds are evident while detecting CST cycles. Excluding trunk pitch angle ($\theta _i$) from classification reduced strategy F1 scores substantially, underscoring its role in distinguishing MT from Flexion. Sensitivity analysis on the amplitude parameter $\alpha$ showed stable performance across the tested range (0.1$-$0.4) (see Table 2).

**Table 2:** Sensitivity analysis of key algorithm components

Configuration	Count accuracy	Cycle time MAE (ms)	Strategy macro F1	ICC(3,1) counts /time
Full model (DF guard + $\theta$, $\alpha {=}0.2$)	0.99	24 (22–27)	0.94	0.95 / 0.95
Without DF guard	0.78	112 (97–128)	0.86	0.81 / 0.84
Without trunk pitch ($\theta$)	0.99	26 (23–30)	0.77	0.95 / 0.95
Without both	0.71	146 (124–168)	0.61	0.78 / 0.80
$\alpha$ sensitivity (0.1 / 0.4)	0.98 / 0.97	28 / 33	0.93 / 0.91	0.94 / 0.93

Values are mean (95% CI) where applicable. Removing the dominant-frequency guard or trunk pitch estimation substantially degrades detection accuracy and strategy classification performance, confirming the importance of these components

## Discussion

Our approach prioritizes transparency and interpretability by employing a fully rule-based framework, offering clear decision logic that contrasts with the opacity often associated with black-box machine learning models. We have detailed the filtering, amplitude adaptation, and dominant-frequency thresholding steps to ensure reproducibility. Sensitivity analysis confirmed their critical role, with removal causing a 21–28% drop in detection accuracy (Table 2). This design ensures that clinicians can understand and trust the decision process, making the approach more suitable for clinical applications where interpretability and reproducibility are essential.

Our findings show that a waist-mounted smartphone is capable of capturing detailed cycle-level metrics during CST, including timing and strategy classification, with high accuracy and reliability. Previous smartphone-based CST systems reported timing errors between 100 and 200 ms [29, 30]. In contrast, our approach achieves substantially lower timing errors, with participant-level MAE on the order of 30–40 ms (group-specific MAE 24–61 ms) and providing biomechanically meaningful strategy labels.

Prior smartphone studies validated CST timing with ICC values around 0.90–0.93 [29], whereas our method achieved ICC$\approx$0.95 across counts, cycle times, and strategy labels. Unlike Cerrito et al. [30] and Cobo et al. [29], which focused on repetition counting, our approach integrates context-aware thresholds and gyroscope-derived trunk pitch to classify MT vs Flexion strategies, an aspect rarely addressed in prior smartphone work.

Compared to Tulipani et al. [42], who used two IMUs and multi-day monitoring to achieve AUC up to 0.89 for fall-risk discrimination in PwMS, our pipeline offers a practical alternative: single device, real time analysis without sacrificing interpretability. While Tulipani’s multi sensor approach enables broader clinical inference, it imposes higher setup complexity and cost.

Flexion cycles were 52–81% longer than MT cycles within each cohort (Table 1), consistent with prior biomechanical studies linking prolonged trunk flexion to reduced strength and postural control [18]. These differences were most pronounced in older adults and participants with PD, aligning with known age- and disease-related mobility constraints.

By quantifying sit-to-stand and stand-to-sit durations, full-cycle time, and trunk pitch angle, our system provides clinicians with actionable metrics for identifying compensatory strategies and tailoring interventions without requiring motion capture or force plates.

Cycle time and rising strategy have been linked to functional status and fall risk in prior studies. Tulipani et al. [42] reported that slower sit-to-stand transitions and altered trunk control were associated with higher fall risk in people with multiple sclerosis (AUC up to 0.89). Similarly, prolonged Flexion strategy use has been observed in frail older adults and individuals with PD, reflecting reduced strength and postural control [18].

The rising strategy detection will help in clinical decision-making. Detailed timing of sit-to-stand and stand-to-sit phases, combined with strategy classification (MT vs. Flexion), can help clinicians tailor interventions for strength and balance training, monitor rehabilitation progress, and detect early compensatory patterns indicative of functional decline. However, such applications should be considered exploratory until confirmed in larger and more diverse samples.

The statistical validation addressed the critical concern regarding non-independence of cycle-level observations by implementing participant-level aggregation and a Linear Mixed Model, ensuring robustness while respecting the nested data structure. High reliability (ICC(2,1) = 0.998) and negligible bias (− 0.012 s) confirm the accuracy of the smartphone-based pipeline. This performance contrasts with prior CST systems reporting timing errors of 100–200 ms. Our approach achieved strong agreement ($ICC(3,1) \approx 0.95$) for cycle times, repetition counts, and strategy labels, exceeding ICC values of 0.90–0.93 reported in earlier smartphone studies.

Sensitivity analysis confirmed the necessity of our design choices (Table 2). Removing the dominant-frequency guard reduced detection accuracy from 99% to 78% and increased cycle time MAE from 24 to 112 ms. Excluding trunk pitch degraded strategy classification (macro F1 from 0.94 to 0.77), underscoring its role in distinguishing MT from Flexion. Removing both components caused the largest performance drop (accuracy 0.71; macro F1 0.61). These findings validate the theoretical rationale for combining frequency-domain constraints with angular kinematics. The amplitude parameter $\alpha$ showed stable performance across the tested range (0.1–0.4), indicating robustness to inter-individual variability.

Our work brings three key contributions. First, it provides automated cycle timing and rising strategy labels using just one smartphone at the waist, with high accuracy across younger, older, and Parkinson’s groups. Second, it uses an adaptive detection method that adjusts to each person and applies a frequency-based check to avoid false counts, which proved essential in our tests. Third, it estimates trunk pitch from short gyroscope windows, avoiding gravity errors and making strategy classification reliable.

Key strengths include (i) end-to-end automation using a smartphone, (ii) cycle-level metrics validated against synchronized video with ICC$\approx$0.95, and (iii) integration of biomechanical features for strategy-aware analysis. This study has several limitations. First, the sample size was modest (n=35) with limited representation of severe mobility impairments, which constrains generalizability. Second, factors such as chair height variability, medication state in participants with Parkinson’s disease, and repeatability of smartphone placement could influence performance metrics. Third, external validity remains to be established under real-world conditions, including unsupervised home use, heterogeneous environments. Unsupervised home use offers individuals the ability to assess their own performance and rising strategies from seated positions beyond standard chairs, such as beds or sofas, which are rarely evaluated in clinical settings. A smartphone-based system enables the capture of natural variations in rising strategies, providing insights into daily functional activities under real-world conditions. This capability has the potential to facilitate personalized feedback and early detection of functional decline or deteriorating mobility, making it a promising avenue for future research and practical implementation. While this remains a limitation of the current study, it aligns with our long-term objective to advance remote monitoring solutions. This study is conducted on a single-smartphone model, which restricts generalizability. Device-specific differences in sensors and operating systems may influence performance, and future work will include testing across multiple devices to enhance robustness. Although our method combines accelerometer and gyroscope features, it does not implement continuous drift correction (e.g., Kalman filtering). Future work will incorporate lightweight fusion for free-living environments.

We developed a single-smartphone framework capable of automated, cycle-level quantification of the CST. Rising strategy classification was achieved through a combination of amplitude and DF adaptive thresholds, and gyroscope-derived trunk pitch, enabling robust detection across varied movement profiles. The system demonstrated high detection accuracy ($\approx$99%), a mean absolute error below 40 ms, and excellent reliability (ICC$\approx$0.95). Flexion cycles were consistently longer than MT cycles, with strategy distribution varying by age and Parkinson’s disease status, reflecting underlying biomechanical demands. This framework offers a practical bridge between laboratory grade motion analysis and scalable, community-based assessment using everyday mobile technology. While our solution shows promise for future clinical integration, the current findings represent an initial feasibility study and should not be generalized to diagnostic or personalized rehabilitation contexts without further evidence from larger, more diverse trials.

Future research will extend validation to longitudinal study with stroke subjects participating in 12-week rehabilitation program, including frail populations and home environments, to assess generalization under real-world variability. Three directions are prioritized: (i) longitudinal monitoring to capture within-subject changes and (ii) predictive modeling that leverages cycle-level features and rising strategy to estimate clinical outcomes, such as fall risk or functional decline. In parallel, the current proof-of-concept software will undergo further refinement and validation in larger, free-living cohorts. Upon completion of this development phase, we aim to release the finalized tools as open-access resources for researchers and clinicians to foster collaboration and accelerate translation into practice.

## Data Availability

Data will be made available on request.
